# Occurrence of Filamentous Fungi and Mycotoxins in Fresh and Minimally Processed Leafy Vegetables from Gardens and Markets

**DOI:** 10.3390/foods15010064

**Published:** 2025-12-25

**Authors:** Ewelina Farian, Katarzyna Kowalczyk, Angelina Wójcik-Fatla

**Affiliations:** Department of Health Biohazards and Parasitology, Institute of Rural Health, Jaczewskiego 2, 20-090 Lublin, Poland; farian.ewelina@imw.lublin.pl (E.F.); kowalczyk.katarzyna@imw.lublin.pl (K.K.)

**Keywords:** leafy vegetables, sprouts, filamentous fungi, mycotoxins, food safety

## Abstract

Fresh, minimally processed foods contain many valuable nutrients but are also a source of pathogenic microorganisms. This study aimed to investigate the presence of filamentous fungi and mycotoxin contamination in leafy vegetables. A total of 160 samples of lettuce, spinach, mixed salads, and sprouts from markets and gardens were tested. Fungal strains were cultured on Malt Extract Agar with chloramphenicol (50 mg/L). Fungal identification was performed by macroscopic and microscopic observations, amplification of the small subunit rRNA (SSU rRNA) gene fragment, and sequencing. Total aflatoxins, aflatoxin B1, and zearalenone contents were determined using the ELISA method. The mean concentrations of filamentous fungi in fresh and minimally processed vegetables were 9.4 × 10^2^ CFU/g and 3.4 × 10^2^ CFU/g, respectively. Nineteen fungal genera were identified, in addition to non-sporulating fungi, of which the largest percentage comprised the genera *Cladosporium* (38%), *Alternaria* (37%), and *Fusarium* (30%), and less frequently *Penicillium*, *Mucor*, *Trichoderma*, and *Aspergillus* (from 8 to 14% of positive samples). The highest percentage of samples contaminated with zearalenone was observed in the spinach group. Ready-to-eat leafy vegetables should be monitored for contamination with filamentous fungi and mycotoxins as they pose a potential risk to consumer health.

## 1. Introduction

The rising interest in healthy food and shifts in consumer lifestyles have led to increased consumption of fresh and easy-to-prepare food products. According to recent reports provided by the Food and Agriculture Organization (FAO), total global spinach production in 2023 was 34.1 million tons, while lettuce production was 28.0 million tons [1]. Leafy green vegetables such as lettuce, spinach, and sprouts possess numerous nutritional properties and nutraceutical compounds. They are a good source of natural dietary fiber, vitamins (C, A, E, K, and niacin), minerals (potassium, magnesium, calcium, phosphorus, and zinc), essential fatty acids, and various bioactive compounds such as polyphenols, carotenoids, and flavonoids [2]. Phytonutrient-rich sprouts and spinach are used to increase the antioxidant capacity and nutritional and functional value of foods consumed worldwide [3,4]. Dietary recommendations suggest consuming approximately 400 g (or five servings) of fruit and vegetables per day, including at least one portion of green leafy vegetables [5,6].

Despite their numerous health benefits, eating raw leafy vegetables carries a potential risk of food poisoning caused by harmful biological agents. Fresh greens are an excellent source of water and nutrients, which are essential for the growth of bacteria and molds. According to previous studies [7], cases of foodborne illness have been linked to poor personal and environmental hygiene, improper food-handling practices, or failure to adhere to recommendations regarding temperature and exposure time. Vegetables can become biologically contaminated at various stages of the production chain, both pre- and post-harvest. Sources of pathogens in vegetables before harvest include contaminated crop soil, irrigation water and fungicide solutions, improperly composted fertilizers, and human and animal interactions with vegetables during cultivation. Post-harvest, plant products can become contaminated during transportation, storage, and irrigation [8,9]. Ensuring food safety, therefore, requires a multifaceted approach at every link in the food chain [10]. Reducing the risk to human health involves not only implementing laws and regulations limiting the possibility of product contamination with biological agents, but also important educational activities for those involved in food production.

Regarding the quality of pre-cut fruit and vegetables (on a ready-to-eat basis), Commission Regulation (EC) No 2073/2005 of 15 November 2005 on microbiological criteria for foodstuffs lists *Salmonella* spp. and *Escherichia coli* bacteria as potential risks [11]. Fungal contamination is closely related to microbiological criteria, which define the permissible limits for these microorganisms. These criteria are designed to ensure food safety and quality, and exceeding them can lead to health risks, such as infections, allergies, or toxicity. Standard ranges have not been developed for other possible biological contaminants, including other bacterial or fungal species. Processing to produce fresh-cut fruits and vegetables can lead to microbiological damage, which, in turn, shortens shelf life [7]. Given the wide range of microorganisms found in fresh and minimally processed foods, determining which species are considered indicators of food microbiological purity is a challenge in the context of changing climatic conditions and the implementation of new production technologies and research methods.

Current knowledge of plant contamination by filamentous fungi and the metabolites they produce remains quite limited [9,12]. In addition to the health of these products, high concentrations of molds and mycotoxins may also indicate improper production conditions or incorrect processing [13], thus providing information on which stages of the food chain should be controlled more rigorously. Previous studies indicate that the most common fungi causing vegetable spoilage include representatives of the genera *Botrytis*, *Alternaria*, *Cladosporium*, *Aspergillus*, *Penicillium*, and *Fusarium*, which can produce mycotoxins [14]. These toxic metabolites exhibit varying levels of toxicity to human health, ranging from allergens, nephrotoxicity, and teratogenicity to carcinogenicity [15,16], which is a well-known threat to public health [17].

Maintaining the microbiological quality of food products is crucial for consumer health and food safety. Illnesses caused by potentially pathogenic filamentous fungi mainly result from the inhalation of conidia, which can cause or exacerbate allergic symptoms. The gastrointestinal tract is also a source of fungal infections and carries an increased risk of mycotoxin exposure [18]. The recommended frequent consumption and lack of heat treatment administered to raw or minimally processed vegetables further increase the risk of negative health effects. Knowledge of mycotoxin concentrations in fresh products is limited, primarily due to their short shelf life and the relatively long time required for mycotoxin testing. For these reasons, most data are derived from analyses conducted on products such as cereals and nuts, which have long storage times [19]. Assessing the risks associated with mycological contamination of ready-to-eat vegetables requires further, more detailed studies. Furthermore, there are no established mycological criteria, including permissible concentration levels, for any mycotoxins for leafy vegetables intended for consumption.

Poland is an important region for microbiological research on vegetables, primarily due to its large-scale vegetable production, the diversity of agricultural practices (conventional and organic), and the need to ensure food safety for the domestic and European markets. The varied climate and environmental conditions across different regions of Poland can create microbiological challenges that demand local research. Moreover, Polish research in this area is limited. Therefore, this study aims to fill knowledge gaps regarding the quality of green vegetables available in this country.

This study aimed to determine the prevalence of filamentous fungi and assess the levels of mycotoxin contamination in fresh and minimally processed vegetables (lettuce, spinach, mixed salads, and sprouts) in the south-eastern region of Poland.

## 2. Materials and Methods

### 2.1. Sample Collection

A total of 160 samples of leafy and minimally processed vegetables were collected in South-Eastern Poland (Lublin region) from May 2024 to March 2025. For each vegetable group, the same number of products were tested. The study was conducted for four product types, including 40 samples each of fresh lettuce and spinach, and ready-to-eat salad mixes and sprouts. The research material came from two sources: vegetable gardens (33 samples: *n* = 28 lettuce and *n* = 5 spinach) and markets (127 samples: *n* = 12 lettuce; *n* = 35 spinach; *n* = 40 salad mix; and *n* = 40 sprout). The sprout group included radish sprouts (*n* = 13), broccoli sprouts (*n* = 9), kale sprouts (*n* = 5), sunflower sprouts (*n* = 5), mung bean sprouts (*n* = 3), alfalfa sprouts (*n* = 2), kale and kohlrabi sprouts (*n* = 1), clover sprouts (*n* = 1), and watercress microleaves (*n* = 1). Availability of sprouts and mixed salads was limited in markets, while lettuce and spinach were sourced directly from both markets and gardens.

Food samples from home gardens were collected in sterile, tightly closed bags, while the products purchased from supermarkets were transported in the manufacturer’s original packaging. All samples were delivered to the laboratory within 2–4 h at a temperature of 4 °C in a portable refrigerator (Campingaz Icetime, Italy) to minimize the risk of fungal proliferation. Immediately upon arrival, samples were prepared, and microbiological cultures were performed.

### 2.2. Culture-Based Identification Methods

The vegetable samples were prepared by suspending 10 g of the sample in 90 mL of Ringer’s solution (Biomaxima, Lublin, Poland) and homogenizing it for 4 min [20] using Bag Mixer 400SW (Interscience, Saint Nom la Bretèche, France). Fungal strains were isolated using the dilution method on Malt Extract Agar (MEA, BD Difco Laboratories, Detroit, MI, USA) with chloramphenicol (PPH Galfarm, Kraków, Poland). Plates were incubated at 30 °C for 72 h (according to the manufacturer’s instructions) and then at 25 °C for another 48 h. The use of two temperatures allowed a larger number of fungal species to be obtained: a temperature of 30 °C is recommended for culturing molds on malt agar, while a temperature of 25 °C [20] is more optimal for the general, vegetative growth of many fungal species. These conditions ensure appropriate colony coloration, facilitating microscopic identification. Fungal concentrations were expressed as the number of colony-forming units per gram of the sample (CFU/g).

The initial identification of fungal species was performed macroscopically (pigmentation and shape and size of the colony) and microscopically (lactophenol staining; presence and appearance of hyphae and spores) using key atlases [20,21].

### 2.3. Molecular-Based Identification Methods

Genomic DNA was isolated from the 72 h culture using Fungi DNA Mini-Kits (Syngen Biotech, Wrocław, Poland), according to the manufacturer’s protocol. DNA purity (value 260/280) was measured using a QIAxpert spectrophotometer (Qiagen, Detroit, MI, USA). The purity of all samples ranged from 1.2 to 3.7; most strains ranged from 1.5 to 2.0. Samples with purity outside this range yielded positive PCR results, but sequencing was unsuccessful or resulted in identification at the genus level.

Detection and species diversity were performed by amplification of a small subunit rRNA (SSU rRNA) gene fragment following the method described by Borneman and Hartin [22]. The presence of *Aspergillus* species in the section *Nigri* was determined according to the method described by Sugita et al. [23]. The negative control for the PCR reactions was nuclease-free water. The positive control was DNA from *A. fumigatus* and *A. niger* strains isolated and confirmed by sequencing in our previous studies [24]. The components and conditions of all PCRs performed in this study are listed in Table S1 (Supplementary Material).

All PCRs were performed using a C1000 Thermal Cycler (BioRad, Hercules, CA, USA) and Mastercycler^®^nexus GSX1 (Eppendorf, Hamburg, Germany). Amplification products were identified in 2% agarose gels (Prona, Basica LE, ABO, Gdańsk, Poland) by horizontal electrophoresis under standard conditions (75 V, 400 mA, 55 min). The amplification products were visualized using a gel documentation and image analysis system with GeneSnap software version 7.12.02 (InGenius LhR, Syngene, Cambridge, UK).

Sequencing was performed by Genomed S.A. (Warsaw, Poland). The obtained sequences were compared to GenBank sequences using the Basic Local Alignment Search Tool (BLAST) (version NCBI BLAST: BLAST+ 2.17.0) and deposited in the GenBank database.

### 2.4. Determination of Mycotoxins

The content of total aflatoxins (B1, B2, G1, G2) (AFT), aflatoxin B1 (AFB1), and zearalenone (ZEA) was determined using the ELISA method and the following sets: Ridascreen^®^ Aflatoxin Total, Ridascreen^®^ Aflatoxin B1 30/15, and Ridascreen^®^ Zearalenon (R-Biopharm AG, Darmstadt, Germany). These tests were performed according to the manufacturer’s instructions using the standards (reference solutions) provided with the sets. Standards at 6 concentrations (0, 0.05, 0.15, 0.45, 1.35 and 4.05 µg/kg) were used to prepare the standard curve for AFT; standards at 6 concentrations (0, 1, 5, 10, 20 and 50 µg/kg) were used for AFB1; and standards at 5 concentrations (0, 50, 100, 200 and 400 µg/kg) were used for ZEA. Standards and samples were analyzed in duplicate. Control validation of tests was performed for Ridascreen^®^ (R-Biopharm AG, Darmstadt, Germany) Aflatoxin B1 30/15, using the analytical standard AFB1 (Trilogy Analytical Laboratory, Washington, DC, USA). The concentrations of the standards used for each type of vegetable, including the mean concentration values and the recovery percentages, are presented in Table S2. The optical density measure was read at a wavelength of λ = 450 nm using a Multiskan FC microplate reader (Thermo Fisher Scientific, Waltham, MA, USA). RIDASOFT^®^ Win.NET software (version 1.9) (R-Biopharm AG, Darmstadt, Germany) was used to create a standard curve and calculate the concentration of mycotoxins in the samples. The limit of detection (LOD) and limit of quantification (LOQ) for each of the tested mycotoxins were determined according to the manufacturer’s recommendations, with the following values: for AFT, the LOD value was <1.75 µg/kg; for AFB1, the value was <1.00 µg/kg; and for ZEA, the value was <0.25 µg/kg.

### 2.5. Statistical Analysis

The results were analyzed using the Mann–Whitney and Kruskal–Wallis tests with STATISTICA v. 5.1 package (Statsoft, Tulsa, OK, USA). The Mann–Whitney test was used to assess differences in fungal contamination between vegetables from gardens and markets and fresh and minimally processed products. The Kruskal–Wallis test was used to compare fungal concentrations across all vegetable groups tested.

## 3. Results

### 3.1. Quantitative Analysis of Fungal Contamination

The presence of filamentous fungi was confirmed in 115 (72%) of the 160 vegetable samples tested (Table 1). The research conducted indicated that the concentration of fungi varied depending on the type of vegetable tested (*p* = 0.018). The highest percentage of positive results was observed in the spinach group, as the presence of filamentous fungi was recorded in 38 out of 40 spinach samples (95.0%). The average concentration of filamentous fungi in spinach samples from gardens was 7.2 × 10^2^ CFU/g, while for samples from markets, it was 1.2 × 10^3^ CFU/g. Similar differences were observed in lettuce samples collected from gardens and markets, with average concentrations of filamentous fungi at 7.4 × 10^2^ CFU/g and 6.1 × 10^3^ CFU/g, respectively. The type of sampling site for leafy vegetables (lettuce, spinach) had no significant effect on the fungal concentration (*p* = 0.66). Statistically significant differences in filamentous fungi concentrations were observed between fresh vegetables (lettuce and spinach) and minimally processed vegetables (mixed salad and sprouts). Notably, higher concentrations of filamentous fungi were found in fresh vegetables (*p* = 0.0033).

Among sprouts, fungal growth was detected in three samples of radish sprouts (mean concentration of 85 CFU/g), two samples of broccoli (620 CFU/g), two samples of kale (35 CFU/g), five samples of sunflower sprouts (542 CFU/g), two samples of alfalfa (1250 CFU/g), and one sample each for mung bean (40 CFU/g) and watercress (1300 CFU/g). The lowest concentration of filamentous fungi was recorded in mixed salads, and the concentrations obtained did not exceed 700 CFU/g.

### 3.2. Generic and Species Composition of Filamentous Fungi

From the entire group of vegetables studied, non-sporulating fungi and 19 genera were identified. Most of the samples tested (160) contained non-sporulating fungi (40.6%), which were detected in 53.8% of the fresh vegetables tested and 27.5% of the minimally processed vegetables. *Cladosporium* (38.1%), *Alternaria* (36.9%), and *Fusarium* (30.0%) were present in slightly smaller percentages. Fungi from the genera *Penicillium*, *Mucor*, *Trichoderma*, and *Aspergillus* were identified less frequently (in 8 to 14% of positive samples). The highest diversity of filamentous fungi was detected in samples of lettuce (14 genera) and spinach (12 genera) (Table 2). The highest number of fungal colonies was isolated from lettuce samples (126/333, 37.8% of all isolated colonies), followed by spinach (108/333, 32.4%) and mixed lettuce (67/333, 20.1%), and the lowest number was obtained from sprout samples (32/333, 9.6%).

**Figure 1:**
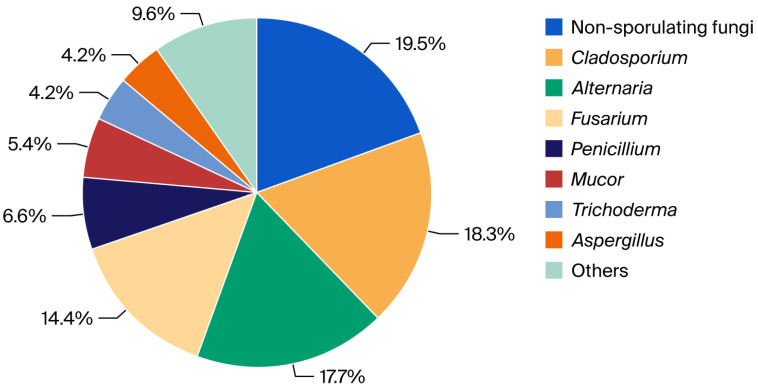
Percentage of non-sporulating fungi and genera identified in the pool of fungal colonies.

Of the 333 fungal colonies collected, the largest percentage was obtained for non-sporulating fungi and the genera *Cladosporium*, *Alternaria*, and *Fusarium*, ranging from 14 to almost 20% (Figure 1).

Molecular analysis confirmed the presence of 13 species of filamentous fungi. All obtained sequences were deposited in GenBank (Table S3). The largest number of sequenced species were fungi isolated from lettuce samples, mainly from the *Fusarium* genus (*F. oxyporum*: PV298050.1, PV298051.1, PV298053.1, PV298061., PV298067.1, and *F graminearum*: PV298052.1), *Mucor* (*M. circinelloides*: PV298068.1, PV298071.1), and *Alternaria* (*A. alternata*: PV298064.1, PV298065.1). *Aspergillus fumigatus* (PV298060.1) and *Aspergillus niger* (PV329690.1) were detected in baby spinach. *Fusarium culmorum* (PV298057.1, PV298058.1) was detected in one spinach sample, and *Trichoderma viride* (PV298069.1) was detected in a sample of mixed salad with spinach.

### 3.3. Co-Occurrence of Filamentous Fungi in Leafy Vegetables

**Figure 2:**
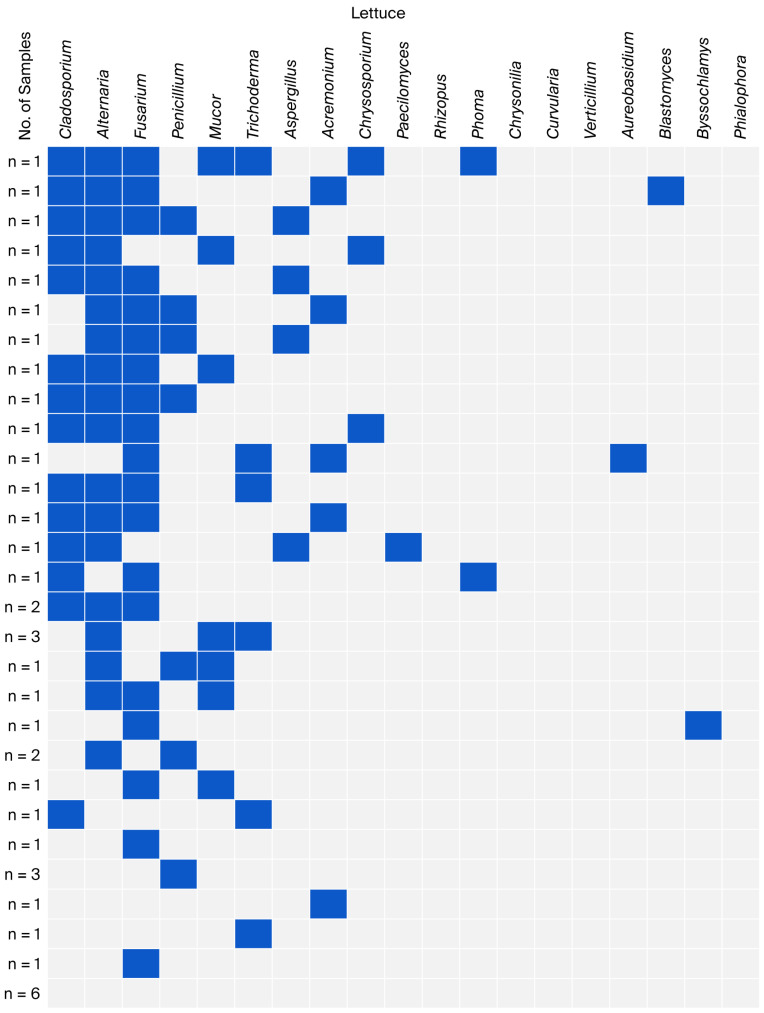
Co-occurrence of fungal genera in lettuce samples.

**Figure 3:**
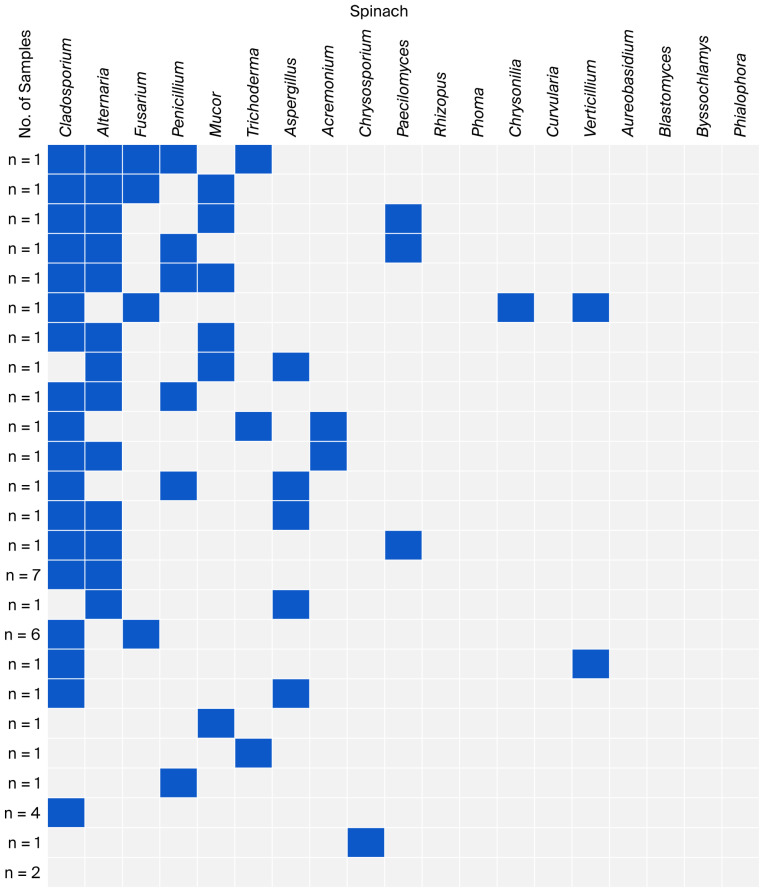
Co-occurrence of fungal genera in spinach samples.

**Figure 4:**
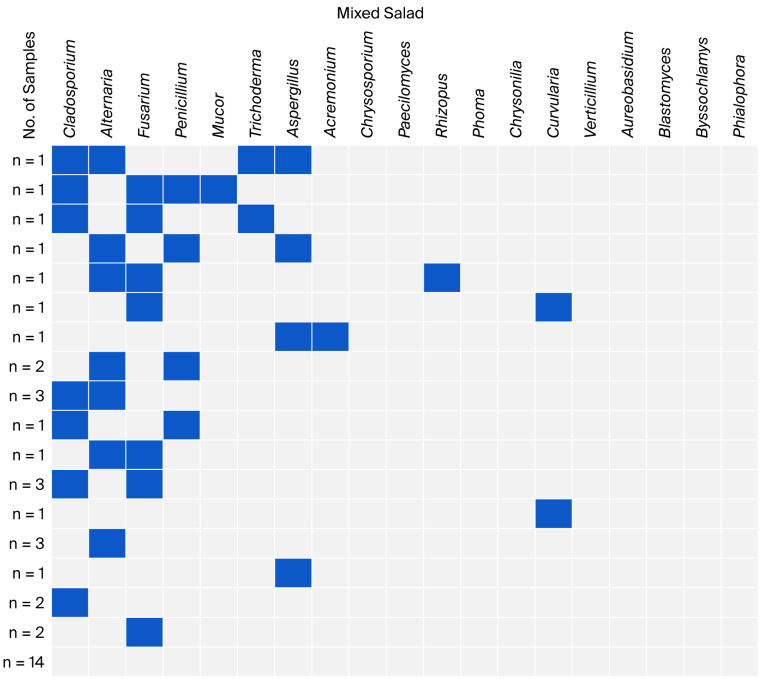
Co-occurrence of fungal genera in mixed salad samples.

**Figure 5:**
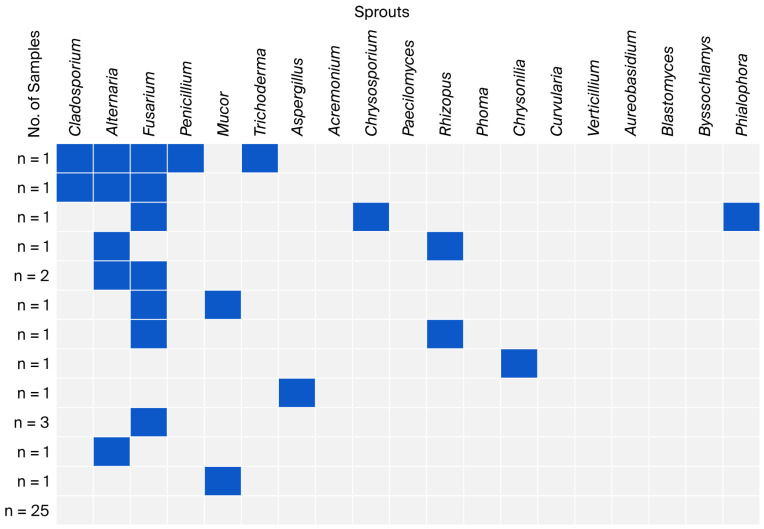
Co-occurrence of fungal genera in sprout samples.

The co-occurrence of fungal genera identified in individual groups of leafy vegetables is presented in Figure 2, Figure 3, Figure 4 and Figure 5. Non-sporing fungi were excluded due to difficulties in their microscopic and macroscopic identification and further analysis on genera or species using molecular methods.

In the group of lettuce samples, the most common co-occurrences were found for the genera *Cladosporium* and *Alternaria* with *Fusarium* (11 samples); *Cladosporium* with *Alternaria* (13 samples); and *Alternaria* with *Fusarium* (14 samples). Over 50% of the samples (22 samples) contained co-occurrences of three to seven fungal genera, with *Alternaria* predominating (20 samples). The remaining samples (12 samples) showed single and double co-occurrences, with *Fusarium* predominating in five samples (Figure 2).

Among the spinach samples, the co-occurrence of *Cladosporium* with *Alternaria* was dominant (*n* = 17). Unlike the lettuce samples, spinach had a lower co-occurrence of fungi from the genus *Fusarium*, especially with *Alternaria* (*n* = 2) (Figure 3).

In the mixed salad samples, only five samples showed co-occurrence of three or four fungal genera. Single and double co-occurrences predominated (over 52%). However, in over one-third of the samples, no fungal genera were identified, and no triple co-occurrence of *Cladosporium*, *Alternaria*, and *Fusarium* was observed (Figure 4).

The highest number of samples without identified fungal genera was found among sprouts (over 62%). Three samples contained three or four genera, including two triple co-occurrences: *Cladosporium*, *Alternaria*, and *Fusarium*. Considering all co-occurrences, the genus *Fusarium* had the highest proportion (*n* = 7) (Figure 5).

### 3.4. Mycotoxin Contamination

The highest percentage of samples contaminated with mycotoxins was observed in the spinach group, where at least one of the tested mycotoxins was detected in 29 samples (72.5%). All spinach samples in which mycotoxins were detected were purchased from stores as ready-to-eat products. The co-occurrence of aflatoxin and ZEA was detected in 9 (22.5%) samples, while ZEA was detected in 20 (50.0%) samples. In the remaining vegetable groups, the presence of mycotoxins was identified in single samples (Table 3).

## 4. Discussion

Current knowledge of the extent of mycological contamination in leafy vegetables is limited, particularly in terms of the contamination limits that can provide consumer protection. The results of the few studies conducted in Poland to date indicate that the level of contamination in fresh, ready-to-eat vegetables appears to be moderate or low. In this study, the presence of filamentous fungi was confirmed in 115 (71.9%) leafy vegetable samples, with mean concentrations ranging from 2.2 log CFU/g in mixed salad to 3.0 log CFU/g in spinach. Other studies conducted in Poland have shown contamination levels of 3.2 log CFU/g and 3.4 CFU/g for spinach, and 3.2 log CFU/g and 2.3 CFU/g for lettuce [25,26]. A lower concentration of 1.2 log CFU/g was found in mixed salads purchased in supermarkets compared to the present study [27].

The range of fungal concentrations in the studies varied from 5 CFU/g to 7.0 × 10^2^ CFU/g, depending on the types of vegetable samples tested. This variation is likely due to changes in environmental conditions, such as temperature and humidity, as well as inconsistencies in food-handling, storage, and processing practices throughout the food chain. Numerous studies cover the range of mycological contamination present from molds and yeasts. The concentrations of these microorganisms in mixed salads vary by country and range from 1.00 to 7.00 CFU/g in Poland [28] and 2.67 to 5.48 log CFU/g in Romania [13] to 0.47 to 8.47 log CFU/g in Argentina [29]. The results obtained by different authors depend on various factors, including local climatic conditions, vegetable varieties, cultivation methods, harvesting, storage, and the research methods used.

In sprouts, filamentous fungi were found in 40% of the samples tested, with an average concentration of 5.1 × 10^2^ CFU/g. In the USA, mycological contamination in sprouts was 4.0 × 10^8^, 2.6 × 10^6^, 6.2 × 10^5^, and 5.2 × 10^6^ CFU/g for beans, alfalfa, broccoli, and crispy sprouts, respectively [17]. In Iran, the average concentration of contaminants in wheat and mung bean sprouts was 6.8 log CFU/g [30]. In both cases, the higher fungal concentration resulted from the combined analysis of filamentous fungi and yeasts. Most microbiological studies on sprouts focus on the identification of bacteria (*Escherichia coli*, *Salmonella enterica*, and *Listeria monocytogenes*) and their potential sources of occurrence (seeds, substrates, and water) [31]. Compared to minimally processed products, fresh vegetables have higher concentrations of filamentous fungi, suggesting that more research is needed to identify possible sources of mycological contamination in seeds, water, and soil.

Over 40% of the microbial community consisted of non-sporulating fungi. Identification using microscopic and macroscopic methods was unsuccessful due to the lack of characteristic phenotypic features that would allow species differentiation. Our study also failed to identify genera and species using molecular methods. Due to the high prevalence of non-spore-forming fungi, more effective methods need to be developed for their identification. This would allow for a better assessment of the health risks posed by fungi in fresh vegetable samples.

Of the 19 identified genera of filamentous fungi in this study, the most common were *Cladosporium*, *Alternaria*, *Fusarium*, and *Penicillium*. The dominance of the genera *Cladosporium* and *Alternaria* was confirmed in leafy vegetables purchased in supermarkets in Italy [32], in fresh vegetables from grocery stores and markets in Slovakia [33], and in ready-to-eat salad purchased from local supermarkets in the USA [17]. In turn, studies conducted in Ghana [34] and Pakistan [35] have shown that *Aspergillus*, mainly *A. fumigatus*, is dominant in ready-to-eat salads. All authors identified virtually the same toxin-producing mold fungi, which are common in the air and can thrive at refrigerated temperatures. Fresh vegetables are typically transported and sold at lower temperatures, which favors fungal growth and mycotoxin production [17]. Cross-contamination in mixed salads cannot be ruled out, especially if they contain leafy greens such as lettuce or spinach, which are in close contact with the soil during growth [33]. By examining the commercially available product only, we cannot assess at what stage contamination occurred—plant growth, initial processing (washing, cutting), packaging, transport, or sale. Human factors, such as poor hygiene practices, also cannot be ruled out. Regardless of region, qualitative studies on the mycobiota composition of green vegetables are consistent, which can help identify the most critical production stages for mold contamination and implement methods to reduce this risk.

Regarding the co-occurrence of fungal genera in leafy vegetable samples, in this study, the co-occurrence of *Cladosporium* with *Alternaria* and *Alternaria* with *Fusarium* was most frequently observed, particularly in groups of lettuce and spinach samples. These results are consistent with studies from Italy, which indicated *Cladosporium* and *Alternaria* as the basic mycobiota of leafy vegetables [32]. *Cladosporium* and *Alternaria* are the most frequently identified fungi in air samples. A meta-analysis of the seasonality of fungal spores in outdoor air, conducted in European regions, found that increasing temperature may be associated with an extended presence of *Cladosporium* and *Alternaria* spores in the air [36]. The second source of potential fungal contamination is seeds. High temperatures may not be sufficiently effective at eliminating fungal species present on the seed surface. Fungal contamination of seeds limits germination but also poses a risk to consumers, for example, in the case of home-grown sprouts [37].

To date, a few studies have indicated the presence of mycotoxins in fresh and minimally processed vegetables. Our study found that over half (72.5%) of spinach samples were contaminated with ZEA. Previous studies have most often identified cereals (corn, oats, and wheat) and cereal products (flour) as a significant source of human exposure to ZEA [38,39]. Only a few studies have confirmed the presence of this mycotoxin in vegetables (red bell peppers and beans) and fruits (banana) [40,41]. To the best of our knowledge, this is the first scientific study confirming the presence of ZEA in spinach. By contrast, AFT was detected in significantly fewer samples (4.4%), and its average concentration was 2.5 times higher than ZEA. Our previous studies have shown that the average AFT concentration in leafy vegetables is higher than in non-leafy vegetables (tomatoes and peppers) [25]. However, in a study conducted in Spain, neither aflatoxins nor ZEA were detected in ready-to-eat vegetables [42].

The main causes of mycotoxin contamination in vegetables are unsuitable environmental conditions, plant damage, and improper storage. Contamination can occur both pre- and post-harvest, even without the presence of fungi. Research by Hariprasad et al. [43] has shown that mycotoxins naturally occurring in agricultural soils are transferred to leafy green vegetables via their root systems. Therefore, monitoring from raw material sources and throughout the stages of processing, packaging, and storage is crucial to ensuring the quality and safety of the finished product. Mycotoxin contamination of food is typically not assessed or regulated, which may lead to an underestimation of its environmental contamination levels. One of the overarching and global documents regarding the regulation of mycotoxins in food and feed is the Codex Alimentarius, specifically Codex Standard CXS 193-1995 (Codex) [44]. However, the Codex only establishes general principles aimed at minimizing mycotoxin contamination in plant materials “as low as reasonably achievable” through the application of Good Agricultural Practices (GAPs) and Good Manufacturing Practices (GMPs), combined with risk assessment.

The European Union (EU) has more detailed and binding legal limits on mycotoxin contamination levels for specific food groups. However, the food products included in the Regulation do not include fresh and minimally processed vegetables. Currently, the content of mycotoxins in selected food products is covered by EU Regulation 2023/915 [45]. The maximum level of ZEA ranges from 20 to 400 μg/kg in foods for infants and young children based on processed cereals and refined corn oil, respectively. The highest concentration of ZEA in this study was 6.1 µg/kg. Although this is below the regulatory limits, frequent consumption of products contaminated with this mycotoxin may negatively affect consumer health. The maximum level of aflatoxins ranged from 4 to 15 μg/kg, respectively, for cereals and their derivatives, dried fruits, peanuts, as well as almonds, pistachios, and apricot kernels.

The European Food Safety Authority (EFSA) has established a tolerable daily intake of 0.25 µg/kg of body weight for ZEA in adults. ZEA is a mycotoxin with strong estrogenic activity that can affect the development and proper functioning of the reproductive and endocrine systems [46]. Therefore, it appears that women may be more susceptible to its effects than men, although this is not confirmed by research. On the other hand, chronic consumption of ZEA in low doses over a long period has been shown to lead to its accumulation in the body with reduced productivity and resistance to pathogens [46,47,48]. Consequently, the presence of ZEA in 82.9% of spinach samples purchased from markets poses a potential risk to consumers due to its established negative health effects (immunotoxic, hepatotoxic, hematotoxic, and estrogenic effects) [49]. The EFSA has not established a tolerable daily intake for AFT, stating only that, due to its genotoxic and carcinogenic effects, its concentration in food products should be as low as possible [50,51]. The International Agency for Research on Cancer (IARC) has classified aflatoxins as human carcinogens (Group 1), demonstrating that their presence in food products may threaten food safety and public health [52].

Fungal growth and mycotoxin production in plant products can be minimized through various preventive measures. Implementing control strategies at specific stages of production can help reduce mycological contamination, which contributes to food spoilage [53]. A key factor in maintaining product quality is avoiding mechanical damage through post-harvest sorting and cleaning. Research conducted by Ibrachim et al. [54] showed that sorting, washing, and drying significantly reduce total aflatoxin contamination in foodstuffs. Conducting heat treatment on leafy vegetables reduces AFB1 levels by 90.5% and 46.5% during pressure-cooking and conventional cooking [43]. Irradiation, ultrasound, and cold plasma also have a significant impact on inhibiting fungal growth and degrading mycotoxins [55,56], while Kong et al. [57] suggest that the use of ozonated water may be an effective control measure. Finally, ozonation of water could be used in the production of leafy vegetables that are consumed raw, such as lettuce, spinach, salad mixes, and sprouts.

### Limitations of This Study

This study provides valuable data on fungal and mycotoxin contamination of leafy vegetables; however, limitations to this study should be noted. A relatively small number of samples were included for each vegetable group in this study, which were harvested or purchased from a single geographic region. Thus, the results do not account for differences in production, storage, and transport conditions due to the unavailability of data, primarily for vegetables purchased in supermarkets. Potential seasonal variations were not analyzed in this study, which may have influenced the quantity and quality of the identified fungal species and genera. Data on crop fertilization may also have been relevant for the analysis of mycotoxin presence.

## 5. Conclusions

The highest concentrations and diversity of filamentous fungi were found in lettuce and spinach samples in close contact with the soil during growth. Higher concentrations were found in fresh vegetables compared to minimally processed products. The most frequently identified species across all sample types, including *Cladosporium*, *Alternaria*, and *Fusarium*, are species capable of growing and producing mycotoxins at low temperatures. The species of *Aspergillus* and *Penicillium* are considered a biological threat to human health.

Mycotoxin contamination was confirmed in all leafy vegetable groups, primarily in spinach. Given the high incidence of ZEA in spinach samples, the routine parallel monitoring of both fungal and mycotoxin contamination is recommended in leafy vegetables.

Despite the limitations of this study, including the relatively small number of leafy vegetable samples examined, the limited regional coverage, and the lack of information on agricultural practices and distribution logistics, this study has significant implications for risk assessment and food safety control measures in the study region. It also provides significant directions for further research. The data presented confirm the need for stricter controls at every stage of food production, especially for ready-to-eat food products. This includes stages such as pre-processing, transport, storage, and sale. The findings point to the need for future global guidelines and regulations regarding the mycological purity of commercially available leafy green vegetables, sprouts, and minimally processed salads.

## Figures and Tables

**Table 1:** Concentration of filamentous fungi in fresh and minimally processed leafy vegetables.

Type of Leafy Vegetables	Prevalence N * (%)	Mean Concentration ± SD ** [CFU/g]	Range [CFU/g]	Interquartile Range [CFU/g]	Median [CFU/g]	Average Concentration [log CFU/g]
Lettuce	34/40 (85.0%)	7.7 × 10^2^ ± 8.1 × 10^2^	5.0–2.6 × 10^3^	85.0–1.1 × 10^3^	5.5 × 10^2^	2.9
Spinach	38/40 (95.0%)	1.1 × 10^3^ ± 1.7 × 10^3^	5.0–6.4 × 10^3^	66.3–1.3 × 10^3^	2.5 × 10^2^	3.0
Mixed salads	27/40 (67.5%)	1.7 × 10^2^ ± 1.9 × 10^2^	5.0–7.0 × 10^2^	45.0–2.0 × 10^2^	65.0	2.2
Sprouts	16/40 (40.0%)	5.1 × 10^2^ ± 5.7 × 10^2^	5.0–1.3 × 10^3^	47.5–1.2 × 10^2^	1.3 × 10^2^	2.7
**Total**	115/160 (71.9%)	7.1 × 10^2^ ± 1.1 × 10^3^	5.0–6.4 × 10^3^	50–0.9 × 10^2^	1.7 × 10^2^	2.9

* N = number of positive samples/number of examined samples (%). ** SD = standard deviation.

**Table 2:** Generic composition of filamentous fungi in fresh and minimally processed leafy vegetables.

Genera of Filamentous Fungi	Type of Samples				Total Occurrence (*n* = 160) N * (%)
	Lettuce (*n* = 40) N * (%)	Spinach (*n* = 40) N * (%)	Mixed Salads (*n* = 40) N * (%)	Sprouts (*n* = 40) N * (%)	
Non-sporulating fungi	24 (60.0)	19 (47.5)	17 (42.5)	5 (12.5)	65 (40.6)
*Cladosporium*	15 (37.5)	32 (80.0)	12 (30.0)	2 (5.0)	61 (38.1)
*Alternaria*	22 (55.0)	19 (47.5)	12 (30.0)	6 (15.0)	59 (36.9)
*Fusarium*	20 (50.0)	9 (22.5)	10 (25.0)	9 (22.5)	48 (30.0)
*Penicillium*	10 (25.0)	6 (15.0)	5 (12.5)	1 (2.5)	22 (13.8)
*Mucor*	9 (22.5)	6 (15.0)	1 (2.5)	2 (5.0)	18 (11.3)
*Trichoderma*	8 (20.0)	3 (7.5)	2 (5.0)	1 (2.5)	14 (8.8)
*Aspergillus*	4 (10.0)	5 (12.5)	4 (10.0)	1 (2.5)	14 (8.8)
*Acremonium*	5 (12.5)	2 (5.0)	1 (2.5)	0 (0.0)	8 (5.0)
*Chrysosporium*	3 (7.5)	1 (2.5)	0 (0.0)	1 (2.5)	5 (3.1)
*Paecilomyces*	1 (2.5)	3 (7.5)	0 (0.0)	0 (0.0)	4 (2.5)
*Rhizopus*	0 (0.0)	0 (0.0)	1 (2.5)	2 (5.0)	3 (1.9)
*Phoma*	2 (5.0)	0 (0.0)	0 (0.0)	0 (0.0)	2 (1.3)
*Chrysonilia*	0 (0.0)	1 (2.5)	0 (0.0)	1 (2.5)	2 (1.3)
*Curvularia*	0 (0.0)	0 (0.0)	2 (5.0)	0 (0.0)	2 (1.3)
*Verticillium*	0 (0.0)	2 (5.0)	0 (0.0)	0 (0.0)	2 (1.3)
*Aureobasidium*	1 (2.5)	0 (0.0)	0 (0.0)	0 (0.0)	1 (0.6)
*Blastomyces*	1 (2.5)	0 (0.0)	0 (0.0)	0 (0.0)	1 (0.6)
*Byssochlamys*	1 (2.5)	0 (0.0)	0 (0.0)	0 (0.0)	1 (0.6)
*Phialophora*	0 (0.0)	0 (0.0)	0 (0.0)	1 (2.5)	1 (0.6)

* N = number of positive samples (%).

**Table 3:** Mycotoxin concentration in fresh and minimally processed leafy vegetables.

Mycotoxins	Mycotoxin Concentration [ng/kg]				
	Average (N *)				Total Average (N *) Range (Values ≥ LOD)
	Lettuce	Spinach	Mixed Salad	Sprouts	
Total aflatoxins	3681.9 (1/40)	2095.3 (5/40)	nd. **	3251.4 (1/40)	2487.1 (7/160) 1752.4–3681.9
Aflatoxin B_1_	nd.	1172.0 (5/40)	nd.	nd.	1172.0 (5/40) 1080.0–1320.0
Zearalenone	nd.	989.1 (29/40)	274.0 (1/40)	274.0 (1/40)	966.8 (31/160) 272.6–6108.3

* N = number of positive samples/number of examined samples. ** nd. = not detected.

## Data Availability

All data related to the current study are available from the corresponding author upon reasonable request. Nucleotide sequences obtained during this study were submitted to the GenBank database (https://www.ncbi.nlm.nih.gov/genbank/; accessed on 22 March 2025) under the accession numbers (PV298049–PV298074 and PV329690–PV329691) and are contained in Table S3 (Supplementary Materials).

## Supplementary Materials

The following supporting information can be downloaded at https://www.mdpi.com/article/10.3390/foods15010064/s1, Table S1. The components and conditions of PCR reactions used to identify filamentous fungi. Table S2. Data on the reliability of measurements of aflatoxin B1 concentration in food products using the ELISA method. Table S3. Fungal genera and species confirmed by sequencing—accession numbers of sequences are deposited in GenBank.
