# Supplementary material for: Occurrence of Filamentous Fungi and Mycotoxins in Fresh and Minimally Processed Leafy Vegetables from Gardens and Markets
Source: Foods. 2025 Dec 25;15(1):64. doi: 10.3390/foods15010064 (PMC12786192; doi:10.3390/foods15010064)
Supplement: Supplementary file 1 [file foods-15-00064-s001.zip › foods-4028793-supplementary.pdf]

Supplementary materials

Table S1. The components and conditions of PCR reactions used to identify filamentous fungi.

Target gene	Amplicon size (bp)	Primer Sequence (5′–3′)		PCR reaction components		PCR Cycling Condition	References
				**Reagents**	**Volume [µl]**		
Major phyla of fungi: *Ascomycota*, *Basidiomycota*, *Chytridomycota*, *Zygomycota*.							
small subunit rRNA (SSU rRNA)	442	1 PCR	nu-SSU-0817: TTAGCATGGAATAATRRAATAGGA<br>nu-SSU-1196: TCTGGACCTGGTGAGTTTCC	10 × PCR buffer (15mM $MgCl_2$)<br>dNTP Mix (2 mM each)<br>Primer each (10 µM)<br>Taq polymerase (5 U/µl)<br>Nuclease-free water<br>Template DNA<br>**Total:**	2.5<br>2.5<br>1.0<br>0.1<br>15.4<br>2.5<br>**25**	Initial denaturation of 2 min at 94 °C followed by 35 cycles:<br>denaturation at 94 °C for 10 s<br>annealing at 56 °C for 15 s<br>extension at 72 °C for 30 s, and final extension at 72 °C for 2 min.	[22]
Aspergillus genus, section *Nigri*							
ITS1	521	1 PCR	ASAP1: CAGCGAGTACATCACCTTGG<br>ASAP2: CCATTGTTGAAAGTTTTAACTGATT	10 × PCR buffer (15mM $MgCl_2$)<br>dNTP Mix (2 mM each)<br>Primer each (10 µM)<br>Taq polymerase (5 U/µl)<br>Nuclease-free water<br>Template DNA<br>**Total:**	2.5<br>2.5<br>1.0<br>0.2<br>15.8<br>2.0<br>**25**	Initial denaturation of 4 min at 94 °C followed by 30 cycles:<br>denaturation at 94 °C for 1 min<br>annealing at 55 °C for 2 min<br>extension at 72 °C for 1.5 min, and final extension at 72 °C for 10 min.	[23]
	310	2 PCR	ASPU: ACTACCGATTGAATGGCTCG<br>Ni1r: ACGCTTTCAGACAGTGTTCG	10 × PCR buffer (15mM $MgCl_2$)<br>dNTP Mix (2 mM each)<br>Primer each (10 µM)<br>Taq polymerase (5 U/µl)<br>Nuclease-free water<br>Template DNA<br>**Total:**	2.5<br>2.5<br>1.0<br>0.2<br>15.8<br>1.0<br>**24**	Initial denaturation of 4 min at 94 °C followed by 25 cycles:<br>denaturation at 94 °C for 1 min<br>annealing at 60 °C for 15 s<br>extension at 72 °C for 15 s, and final extension at 72 °C for 10 min.	

Supplementary materials

Table S2. Data on the reliability of measurements of aflatoxin B1 concentration in food products using the ELISA method.

Type of sample	Standard concentration [µg/kg]	Mean concentration [µg/kg]	Recovery [%]
Lettuce	5	4.8	96.4
	10	8.7	86.1
	20	19.6	98.4
	50	48.7	97.4
Spinach	5	5.3	106
	10	10.1	101
	20	20.3	101
	50	44.7	89.6
Mixed salad	5	4.0	80
	10	9.1	91
	20	20.4	102
	50	46.3	92.6
Sprouts	5	5.4	108
	10	9.5	95
	20	17.4	87
	50	52.0	104

Supplementary materials

Table S3. Fungal genera and species confirmed by sequencing –accession numbers of sequences deposited in GenBank.

	Species confirmed by sequencing	Sample type	Accession number
1	*Acremonium* sp.	lettuce	PV298055.1
2	*Acremonium* sp.	spinach	PV298063.1
3	*Alternaria alternata*	romaine lettuce	PV298064.1
4	*Alternaria alternata*	romaine lettuce	PV298065.1
5	*Aspergillus fumigatus*	baby spinach	PV298060.1
6	*Aspergillus niger*	baby spinach	PV329690.1
7	*Aspergillus niger*	salad mix	PV329691.1
8	*Cladosporium cladosporioides*	romaine lettuce	PV298072.1
9	*Cladosporium* sp.	iceberg lettuce	PV298073.1
10	*Fusarium culmorum*	spinach	PV298057.1
11	*Fusarium culmorum*	spinach	PV298058.1
12	*Fusarium graminearum*	lettuce	PV298052.1
13	*Fusarium oxysporum*	lettuce	PV298050.1
14	*Fusarium oxysporum*	lettuce	PV298051.1
15	*Fusarium oxysporum*	lettuce	PV298053.1
16	*Fusarium oxysporum*	lettuce	PV298061.1
17	*Fusarium oxysporum*	lettuce	PV298067.1
18	*Fusarium* sp.	lettuce	PV298062.1
19	*Mucor circinelloides*	lettuce	PV298068.1
20	*Mucor circinelloides*	lettuce	PV298071.1
21	*Paecilomyces variotii*	baby spinach	PV298070.1
22	*Penicillium chrysogenum*	lettuce	PV298049.1
23	*Penicillium decumbens*	baby leaves mix	PV298074.1
24	*Penicillium oxalicum*	cut salad mix with lamb's lettuce	PV298059.1
25	*Phoma* sp.	lettuce	PV298054.1
26	*Plectosphaerella* sp.	lettuce	PV298056.1
27	*Plectosphaerellaceae* sp.	romaine lettuce	PV298066.1
28	*Trichoderma viride*	mixed salad with spinach	PV298069.1
